# Efficient p*K*_a_ Determination in a Nonaqueous Solvent Using Chemical Shift Imaging

**DOI:** 10.1021/acs.analchem.2c00200

**Published:** 2022-05-27

**Authors:** George Schenck, Krzysztof Baj, Jonathan A. Iggo, Matthew Wallace

**Affiliations:** † Department of Chemistry, University of Liverpool, Liverpool L69 7ZD, U.K.; ‡ School of Pharmacy, University of East Anglia, Norwich Research Park, Norwich NR4 7TJ, U. K

## Abstract

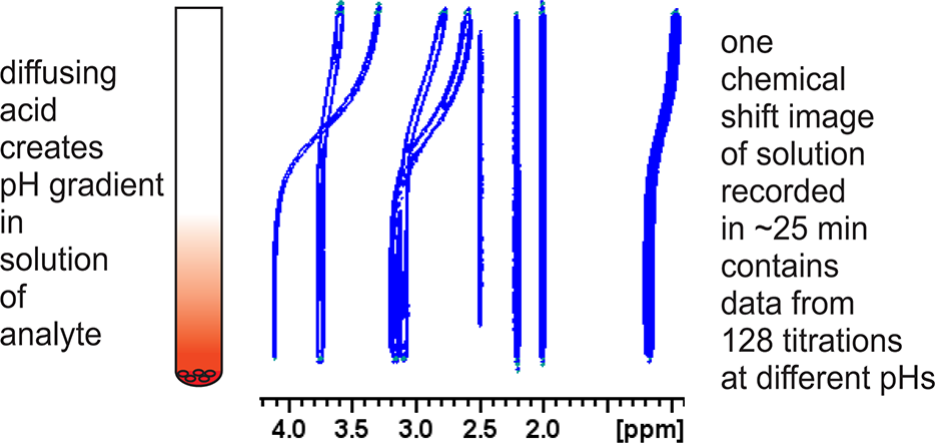

p*K*_a_ is an important property of a molecule which impacts many fields, such as drug design, catalysis, reactivity, and environmental toxicity. It is often necessary to measure p*K*_a_ in nonaqueous media due to the poor solubility of an analyte in water, for example, many compounds of pharmaceutical interest. Although NMR methods to measure p*K*_a_ in water are well established, determining p*K*_a_ in organic solvents is laborious and problematic. We present an efficient one-shot method to determine the p*K*_a_ of an analyte in an organic solvent in a single measurement. Diffusion of an acid into a basic solution of the analyte and a set of pH indicators establishes a pH gradient in the NMR tube. The chemical shift of a pH sensitive resonance of the analyte and the pH of the solution are then determined simultaneously as a function of position along the pH gradient by recording a chemical shift image of the NMR tube. The p*K*_a_ of the analyte is then determined using the Henderson–Hasselbalch equation. The method can be implemented in any laboratory with a gradient equipped NMR high-field spectrometer and is demonstrated for a range of pharmaceutical compounds and inorganic phosphazene bases.

## Introduction

The reactivity, conformation,^1,2^ solubility,^3^ and toxicity in the environment^4^ of a molecule can all be influenced by its protonation state. The acid dissociation constant, normally reported as its negative logarithm, p*K*_a_, is therefore an important and widely used parameter in catalysis, drug design pharmacology, and chemical synthesis.^3,5^ p*K*_a_ is also a vital parameter in proton transfer reactions, self-assembly,^6^ and host guest chemistry.^7^ Acid/base catalysis is also used extensively in industrial processes.^8−10^

We recently described a one-shot procedure for the determination of p*K*_a_ in aqueous solution that allows a complete NMR titration to be performed in a single measurement on a single sample in which a pH gradient has been established.^11^ The method eliminates the labor-intensive preparation of multiple samples of known pH required by traditional NMR based methods by recording a spatially resolved NMR spectrum of a solution of the analyte containing appropriate pH indicators. It is highly efficient both in quantity of sample and of instrument and operator time required.

However, many active pharmaceutical ingredients and drug candidates are too insoluble in water for the precise determination of their p*K*_a_ in aqueous solution.^12,13^ Extension of aqueous methods for p*K*_a_ determination to nonaqueous p*K*_a_ measurements adds another degree of difficulty. For example, standard pH electrodes that have been designed for potentiometric determinations in aqueous media degrade rapidly in nonaqueous solvents,^14^ and pH standard solutions in nonaqueous media are not readily available. There is, therefore, significant interest in^15,30,31^ and need to determine p*K*_a_ in nonaqueous media.^5,16−18^

In this paper, we extend our method to determinations in the biologically relevant p*K*_a_ range in DMSO, an organic solvent commonly used in drug design when determining the p*K*_a_ of poorly water-soluble drug candidates. The method can, in principle, easily be adapted to other organic and mixed solvent systems and can be implemented in any laboratory with access to a gradient equipped NMR spectrometer. Our sequences can be run under automation using robotic sample changers.

## Experimental Section

Experiments were performed in Norell 502 NMR tubes on a Bruker AV-I 400 operating at 400.05 MHz for ^1^H equipped with a Bruker SampleJet sample changer using a Bruker QNP ^1^H-^19^F,^31^P,^13^C probe or manually on an AV-II 400 spectrometer operating at 400.20 MHz for ^1^H using a Bruker TBI ^1^H,^31^P;BB probe. Both spectrometers are equipped with a Bruker GRASP II gradient spectroscopy accessory including a CCU board and 10 A single channel current amplifier for a gradient strength up to 50 G/cm. In total, 16 dummy scans were used. A total of 128 increments and 8 scans per increment were acquired with an acquisition time of 1 s and a recycle delay of 0.1 s giving a total image acquisition time of 20 min.

The solid acids used were saccharin, Meldrum’s acid, barbituric acid, salicylic acid, niacin, aspirin, and 2,4-dinitrobenzoic acid, all obtained from Alfa Aesar. Experimentally determined limiting shifts and literature p*K*_a_s of the indicators used are given in Table S1. Literature p*K*_a_s of acids used are given in Table S2. Experimentally determined p*K*_a_s of the indicators used are given in Table S4. The p*K*_a_ is then determined by least-squares fitting of the data to eq 2, with p*K*_a_, δ_H_, and δ_L_ as free variables. All solutions were prepared using anhydrous DMSO as 5 mL stock solutions in a N_2_ purged glovebox. To establish a pH gradient, 2–10 mg of solid acid was placed into the NMR tube and covered with four glass beads. The acid used in each titration is given in the Supporting Information (Sections S5 and S6, Tables S4–10, and titration curves 1–22).Modified Henderson–Hasselbalch Equation
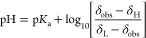
1δ_obs_ is the observed chemical shift and δ_H_ and δ_L_ are the fully protonated and fully deprotonated chemical shifts, respectively.Rearranged Henderson–Hasselbalch Equation
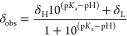
2 Provided that the amount of acid is in slight excess, this was found to have no effect on the precision of p*K*_a_^det^ (section S9). The analyte solution was then carefully layered over the beads and the NMR tube placed in the spectrometer sample changer for the gradient to develop.

The sample changer was not temperature-controlled; the lab temperature was maintained between 18 and 20 °C. This temperature variation had no observable effect on the measurement. The spectrometer probe temperature during collection of the image was maintained at 25 ± 0.2 °C using a Bruker BVT3000 controller. Temperature equilibration occurred during the sample shimming and determination of the water solvent suppression frequency. All measurements were performed in at least duplicate over several months. Furthermore, several images were collected on single samples left in the spectrometer up to 32 h indicating that the precision of the measurements were not affected by differences in gradient equilibration temperatures or temperature equilibration times.

The high viscosity of DMSO solutions means that diffusion is slow; this is advantageous since it results in a wide time window during which useful images can be recorded. Typically, in DMSO useful gradients will be present from 8 to 32 h after addition of the analytical solution, with the optimal time being between 16 and 24 h (see section S8). Results in this paper were recorded after 20–24 h diffusion. Since the total acquisition time of an image is relatively short (∼20–30 min), if desired, the sample can be repeatedly removed from the spectrometer to the autosampler carousel, then replaced in the spectrometer after a further time interval for gradient development and remeasured to ensure an optimal image is obtained. An automation script is included in the Supporting Information.

Images were recorded using the phase encoded pulse sequence of Luy,^19^ adapted to include WATERGATE suppression of residual solvent resonances^20^ to yield a ^1^H NMR spectrum every 0.2 mm along the NMR-active region of the sample. A typical pulse sequence is included in the Supporting Information. A phase encoding gradient pulse of ∼242 μs was used and varied in strength from −27 to 27 G cm^–1^ in 128 increments. Time domain data files were transformed without zero-filling using sine bell apodization. A 128 slice CSI experiment had a total acquisition time of 20 min.

Chemical shift indicators, compounds of known p*K*_a_ that exhibit a change in δ_obs_ with changing pH, are used to determine the solution pH in NMR titrations using the modified Henderson–Hasselbalch equation, eq 1. Protonated (δ_H_) and deprotonated limiting shift (δ_L_) of the indicator compounds were confirmed by independent measurements in acidified and basic DMSO solutions (sections S3 and S7). All indicators and analytes were assumed to be in fast exchange and were confirmed by observation of each as a single continuous sigmoidal trace in the image. Slow exchanging species display a noncontinuous trace which may still be used if the two peaks can be integrated and averaged, though likely at the cost of some precision in the determination. The tracked resonances of analytes and indicators were selected on the basis of sensitivity to pH and presence in an uncrowded spectral region to minimize overlap with other resonances.

The solution pH at each position along the sample is obtained from the chemical shifts of the NMR pH indicators and eq 1. A plot of δ_obs_ of the analyte against pH of the solution yields the titration curve of the analyte, section S6, titration curves 1–22. Typically, 60–80 useful data points for pH and p*K*_a_ determination are obtained from an image.

Ackerman et al. have reported a detailed description of error determination in NMR titrations (eq 3).^21^ The uncertainty in the determination of p*K*_a_ arises primarily from the uncertainty in the limiting chemical shifts and in the p*K*_a_ values of the pH indicators used. The error in each pH measurement for an individual indicator, Δp*K*_a_, far outweighs the second and third terms in our determinations (Δδ_H_ and Δδ_L_ ≈ 0.001 ppm). A minimum of two indicators are used in each titration; the error for each pH point is then the weighted average of the indicator errors. Error varies through the titration; the largest magnitude error in the indicators’ p*K*_a_ has therefore been used as the error in the analyte p*K*_a_ (±0.1 for all titrations).
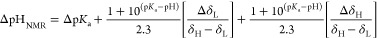
3The error in a single pH point in an NMR titration depends on the precision with which the p*K*_a_ of the indicator is known and the precision of the measured chemical shift differences.

## Results and Discussion

DMSO is widely used as an alternative solvent to water and is often the solvent of choice for medicinal chemists engaged in high-throughput screening of drugs and druglike molecules. It is a relatively inert polar aprotic solvent that can support a wide pH range: DMSO has been used to determine the acidities of trifluoromethanesulfonic acid (0.3)^22^ and diphenylmethane (32.2).^23^ DMSO can also be a more pharmacologically relevant solvent compared to water as it better replicates the lipophilic interior of membranes, which drug molecules must penetrate to reach their target.^16^

DMSO is hygroscopic; therefore, contamination of the sample with water must be considered. Table 1 compares the p*K*_a_ of benzylamine and imidazole determined using our one-shot method in strictly anhydrous DMSO and in solutions to which 1% and 2% H_2_O has been added deliberately. No significant effect of water up to 2% by volume is seen, with differences in the values obtained for p*K*_a_ all being less than the quoted error, demonstrating the utility of the method in a real laboratory setting.

To test the precision of the one-shot method in DMSO, the p*K*_a_s of four basic indicators and two benzoic acids of known p*K*_a_ were determined and compared with the literature.^24^

**Table 1:** p*K*_a_ Determined in Anhydrous DMSO and in 1% Water and 2% Water DMSO Solutions

analyte	p*K*_a_^det^ (±0.1) anhydrous	p*K*_a_^det^ (±0.1) 1% water	p*K*_a_^det^ (±0.1) 2% water
benzylamine	9.78	9.80	9.79
imidazole	6.46	6.40	6.44

The NMR pH indicators used in the determinations were selected according to four criteria: a known p*K*_a_ at a useful point in the pH scale, a proton with an observable ^1^H chemical shift change between δ_L_ and δ_H_, a simple NMR spectrum that does not obscure the resonances of the analyte, and finally the indicators must not react with the analyte other than via proton dissociation. Each analyte was matched with a pair of appropriate pH indicators, and the NMR samples allowed to stand in the NMR autosampler while the pH gradient developed. In all cases, Δp*K*_a_^lit^, the differences between our determinations and the literature, is negligible, Table 2.

**Table 2:** p*K*_a_ in DMSO (p*K*_a_^det^), Indicators and Acids Used, and the Difference from Literature Values (Δp*K*_a_^lit^) for the Analytes

analyte	p*K*_a_^det^ ± 0.1	Δp*K*_a_^lit^	indicators	acid
1-methylimidazole	6.16	+0.01^26^	a, b	i
morpholine	9.01	+0.07^27^	c, d	j
benzylamine	9.78	–0.03^28^	e, f	k
diethylamine	10.42	+0.02^29^	g, f	k
2,4-dinitrobenzoic acid	6.51	–0.01^30^	h, d	l
salicylic acid	6.78	–0.02^31^	h, b or b, d	l
aspirin	8.68		d, c	l
niacin	8.60		d, c or c, g	l
imidazole	6.46	–0.53 to +1.36,^32,26,33,34^	a, h	i

a 2,6-Lutidine.^35^

b Imidazole.

c Triethylamine.^31^

d Dimethylbenzylamine.^36^

e Diethylamine.^29^

f Pyrrolidine.^33^

g Benzylamine.^28^

h 1-Methylimidazole.^26^

i Saccharin.

j Meldrum’s acid.

k Barbituric acid.

l The analyte was used as the diffusing acid.

The p*K*_a_s of niacin and aspirin, for which no literature data in DMSO is available, were next determined. Two sets of indicators (dimethylbenzylamine and triethylamine and triethylamine and benzylamine, Table 2) were used in separate titrations of niacin to test the precision obtainable if a less than optimal choice of pH indicator is made. Despite the insensitivity of the benzylamine reporter resonances at a low pH, the results of the two titrations are in good agreement, showing that even with a single indicator, precise p*K*_a_ data can be obtained (section S6, titration curves 18 and 19). The most similar compound to aspirin for which p*K*_a_ data in DMSO is available is 2-acetamidobenzoic acid (p*K*_a_ = 8.2 ± 0.1)^25^ in which the conjugate base is stabilized by an intramolecular hydrogen bond between the carboxylate and the amide proton. Deprotonation of 2-acetamidobenzoic acid is therefore expected to be more favorable than deprotonation of aspirin which does not possess a stabilizing internal hydrogen bond in its conjugate base. This expectation is borne out by our determined p*K*_a_ of aspirin (8.68 ± 0.1).

A large range of values (5.1–6.94) has previously been reported for the p*K*_a_ of imidazole (p*K*_a_^imid^) in DMSO. By our method we obtain a value of 6.46 ± 0.1, last row, Table 1. To further test the reliability and precision of our method, we have redetermined the p*K*_a_s of 1-methylimidazole and salicylic acid using imidazole as the pH indicator. Table 3 compares the values for the p*K*_a_ of 1-methylimidazole and salicylic acid obtained using our value and each value reported in the literature for p*K*_a_^imid^. Excellent agreement between the p*K*_a_s of the two analytes and their literature values is only obtained using our value of p*K*_a_^imid^ = 6.46. This consistency gives us confidence both in the precision of the method and in our determination of p*K*_a_^imid^.

**Table 3:** Comparison of p*K*_a_s of 1-Methylimidazole and Salicylic Acid Determined Using the Various Literature Values for p*K*_a_^imid^

1-methylimidazole^a^		salicylic acid^b^		imidazole
determined p*K*_a_^det^^c^	Δp*K*_a_^d^	determined p*K*_a_^det^^c^	Δp*K*_a_^d^	reported p*K*_a_^imid^
4.83	1.32	5.14	1.66	5.1 ± 0.2^32^
6.00	0.15	6.60	0.2	6.26 ± 0.06^26^
6.10	0.05	6.64	0.16	6.37 ± 0.04^33^
6.16	–0.01	6.78	0.02	6.46^e^
6.63	–0.48	7.05	–0.25	6.94 ± 0.06^34^

a p*K*_a_ = 6.15.^26^

b p*K*_a_ = 6.8.^31^

c p*K*_a_^det^ calculated using p*K*_a_^imid^.

d p*K*_a_^det^, literature value.

e This work.

Ionic strength is known to influence the observed p*K*_a_. It is usual therefore to conduct p*K*_a_ titrations at constant ionic strength and extrapolate to infinite dilution using a correction to obtain a thermodynamic p*K*_a_.^37^ In a one-shot titration, such corrections are potentially problematic since the concentration of the charged species varies continuously throughout the solution. Fortuitously, using the thermodynamic p*K*_a_ values of the indicators is expected to return the thermodynamic p*K*_a_ values of the analyte directly, provided the analyte and indicators have the same charges as each other in their protonated/deprotonated states.

This is because the Davies-type correction for ionic strength considers only the charge and number of charged species present which does not change. In this situation, the corrections required to the p*K*_a_ of the indicator and to the p*K*_a_ of the analyte cancel (see the Supporting Information section S4). Inspection of Table 2 reveals that, in practice, accurate p*K*_a_ values are returned for both the amine and carboxylic acid analytes studied. We conclude that, at the ionic strengths encountered in this work in DMSO (0.01 to 0.05 M), formal ionic strength corrections are not required. The ionic strength at the midpoint of each titration is provided in the Supporting Information (section S5, Tables S4–S10) since the p*K*_a_ determination by NMR is most sensitive to ionic strength where pH ≈ p*K*_a_.^21,38^

To probe the effect of higher ionic strengths, titrations to determine the p*K*_a_s of morpholine and 1-methylimidazole were performed with 0.1 and 0.2 M LiCl background electrolyte, Table 4. These analytes possess the same charges upon protonation/deprotonation as the indicators and so their p*K*_a_ values are affected equally by ionic strength. No appreciable effect on the determined p*K*_a_ was observed, with the variance in p*K*_a_ being less than 0.1 p*K*_a_ units (0.06). IUPAC guidelines^39^ confirm that NMR titrations with variable ionic strength are acceptable if chemical shifts are shown to be unaffected by ionic strength, which is consistent with the findings of Tynkkyen et al.^38^ and Wallace et al.^11^ We have confirmed that the limiting shifts of 2,6-lutidine, dimethylbenzylamine, and triethylamine are unaffected by ionic strength (section S7).

**Table 4:** Comparison of the p*K*_a_ Determined by the One-Shot Method in DMSO Solution with No Background Electrolyte, 0.1 M LiCl, and 0.2 M LiCl

analyte	p*K*_a_^det^ (±0.1) no electrolyte	p*K*_a_^det^ (±0.1) 0.1 M LiCl	p*K*_a_^det^ (±0.1) 0.2 M LiCl
morpholine	9.01	9.03	8.97
1-methylimidazole	6.16	6.14	6.17

The method is not limited to compounds of pharmaceutical interest but is also effective in determining the p*K*_a_ of compounds outside the biological p*K*_a_ range.

**Figure 1:**
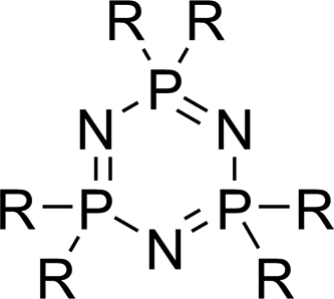
Hexa-aminocyclotriphosphazene. See Table 5 for substituent R.

Cyclotriphosphazenes, Figure 1, are water insoluble inorganic bases that have first p*K*_a_s spanning the range 4–12 which find application as phase transfer catalysts for reactions in organic solvents^40^ and as building blocks for supramolecular assemblies.^41^

Protonation of cyclotriphosphazenes occurs exclusively at the ring nitrogens.^42^ The mono- and diprotonation reactions show distinct chemical shift changes allowing p*K*_a_^1^ = 11.65 and p*K*_a_^2^ < 4 of IPPN to be studied.

Feakins et al. found the second protonation of these bases to be considerably less favorable than the first, p*K*_a_^1^ being around 10 units greater than p*K*_a_^2^ in nitrobenzene.^43^ In this work, the acidic diffusants were chosen to avoid multiple protonation and to span as narrow a pH range as possible, allowing us to focus on p*K*_a_^1^, Table 5. The p*K*_a_s of IPPN and hexa-benzylamino cyclotriphosphazene (BnPN) follow the trend in basicity identified by Feakins,^43^ i.e., that hexa-amino cyclotriphosphazenes have similar basicity to the parent amine. Hexa-morpholino cyclotriphosphazene (morphPN) however does not follow this trend, having a much lower p*K*_a_ than morpholine (4.22 vs 8.94). This may be due to steric blocking of the phosphazene ring nitrogen protonation sites by the morpholine rings which do not have the flexibility of the pendant benzyl or isopropyl groups in IPPN and BnPN.

**Table 5:** p*K*_a_ Values for Three Hexa-amino Cyclotriphosphazenes and the Indicators and Acid Used in Each Titration

analyte	p*K*_a_^det^ (±0.1)	indicators	acid
IPPN (R = NHiPr)	11.65	dea, pyr	barbituric
BnPN (R = NHBn)	9.80	mor,^a^ dea	barbituric
morphPN (R = N(CH_2_CH_2_)_2_O)	4.22	lut, izl	saccharin

a Morpholine.^27^

## Conclusions

An efficient one-shot NMR titration method for p*K*_a_ determination in DMSO has been shown to be applicable to acids and bases. The method gives precise results, agreement with literature values being within ±0.1 p*K*_a_ units for both acidic and basic analytes. The method is robust and insensitive to ionic strength (up to 0.2 M) and water contamination (up to 2%) and opens the door to similar p*K*_a_ determination methods in other nonaqueous and mixed solvent systems. The method has wide applicability and is already being adopted in commercial drug discovery programs.

## Supporting Information Available

Sample changer routines and pulse programs (ZIP)
Experimental details, Henderson–Hasselbalch plots and data tables for all titrations, and discussion of correction for ionic strength (PDF)

## Notes

Raw experimental data is available at DOI: 10.17638/datacat.liverpool.ac.uk/1678.
