# Supplementary material for: Efficient pKa Determination in a Nonaqueous Solvent Using Chemical Shift Imaging
Source: Anal Chem. 2022 May 27;94(23):8115–9. doi: 10.1021/acs.analchem.2c00200 (PMC9201807; doi:10.1021/acs.analchem.2c00200)
Supplement: Supplementary file 1 — ac2c00200_si_001.zip [file ac2c00200_si_001.zip › ReadME.rtf]

Notes
Acquisition tested and working on Bruker AV1-400 with Sample Jet running TopSpin 2.1 pl8 on Windows 10 21H2

Peak picking tested using TopSpin 4.1.3; uses features not included in TS2. 

Installation
Expand ZIP containing all pulse programmes, parameter sets and automation scripts and copy the directory tree to the TopSpin directory
Set USERA1 to chosen user e.g. J.Bloggs
Set USERA2 to data directory root e.g.D:(no final back slash)

Create IconNMR experiments for users
Experiment is CS1_1D_AV1_AC
Set IconNMR options, minimum required are
USERA1 - user who owns the data
USERA2 - data directory root
CNST 63 (default is 0, no solvent suppression)

Data collection
Select experiment CS1_1D_AV1_AC from IconNMR list
Confirm USERA1 and USERA2 - essentially where the data will be stored 
Select CNST63 = 0 (no solvent suppression) or 1 (water suppression using excitation sculpting) using  the IconNMR "user param" button
Submit experiment
If all is working a chemical shift image of the sample will be collected, fourier transformed and phase corrected.

Data processing

Peak picking
Requires manual input of peak picking ranges for the pH indicators used and for the analyte reporter protons

Run au script CSI_peak_pick_AC to generate peak lists for each pH indicator and analyte reporter
The script asks for: 
·	The number of analyte reporters (max 2)and indicators (max 3). HDMS or similar reference TMS type d calibrant is also picked.
·	The location of the fourier transformed and phase corrected 2D dataset - expno and procno
·	The peak picking ranges for the indicators and analyte. HDMS if present is assumed to be in the range 0.5 to -0.5 ppm. If a different calibrant is used, edit line 17 of the au script to suit
·	The peak picking parameters

A peaklist is produced for each indicator and analyte in the experiment root directory.

pH and pKa determinations
Copy-paste the peaklist.txt files into the appropriate cells in the analysis spreadsheet. Indicator and analyte peak positions are re-referenced to experimentally determined HMDS = 0 ppm in each slice. 
The pH of each slice is computed from the referenced chemical shifts of the indicators and their limiting shifts. Two indicators are normally used and a sensitivity weighted average pH calculated for each slice. 
The referenced chemical shift of the analyte at each pH can then be exported to the user's chosen software to extract the analyte pKa.
