# Supplementary material for: Efficient pKa Determination in a Nonaqueous Solvent Using Chemical Shift Imaging
Source: Anal Chem. 2022 May 27;94(23):8115–9. doi: 10.1021/acs.analchem.2c00200 (PMC9201807; doi:10.1021/acs.analchem.2c00200)
Supplement: Supplementary file 2 — ac2c00200_si_002.pdf [file ac2c00200_si_002.pdf]

Supporting Information for

Efficient pK_a Determination in a Non-Aqueous Solvent using Chemical Shift Imaging

George Schenck, Jonathan A. Iggo* and Matthew Wallace†

Department of Chemistry, University of Liverpool, Liverpool L69 7ZD, U.K.

†School of Pharmacy, University of East Anglia, Norwich Research Park, Norwich NR4 7TJ, U. K.

* Email: iggo@liverpool.ac.uk

Supporting Information

Supporting Information *1*

S1- Data Point Selection 2

S2- Niacin and Aspirin pK_a in DMSO 3

S3- Indicator Data 4

Table S1. Independently measured limiting shift and literature pK_a data for basic indicators used in this work 4

Table S2. Literature pKa data for acids used in this work 4

S4- Automatic correction of the analyte pKa when the indicator and analyte have the same charges 5

S5- Experimental details 6

Table S3. Tracked resonances of indicators and analytes in this work 7

Table S4. Experimental details for indicator titrations in DMSO solution 8

Table S5. Imidazole titrations with different masses of saccharin 9

Table S6. Time resolved imidazole titration against 4.2 mg of saccharin 9

Table S7. Experimental details for titrations with acidic analytes 10

Table S8. Experimental details for titrations with phosphazenes 11

Table S9. Experimental details for titrations with background LiCl electrolyte 11

Table S10. Experimental details for titrations with water doped DMSO 12

S6- Titration Data 13

1) Imidazole (anhydrous) 13

2) Imidazole 1% water 13

3) Imidazole 2% water 14

4) 1-methylimidazole 14

5) 1-methylimidazole 0.1M LiCl background electrolyte 15

6) 1-methylimidazole 0.2M LiCl background electrolyte 15

7) Morpholine 16

8) Morpholine 0.1M LiCl background electrolyte 16
9) Morpholine 0.2M LiCl background electrolyte 17
10) Benzylamine (Anhydrous) 17
11) Benzylamine 1% Water 18
12) Benzylamine 2% Water 18
13) Diethylamine 19
14) 2,4-dinitrobenzoic acid 19
15) Salicylic acid (1-methylimidazole and imidazole) 20
16) Salicylic acid (imidazole and dimethylbenzylamine) 20
17) Aspirin 21
18) Niacin (2 indicators- dimethylbenzylamine and triethylamine) 21
19) Niacin (1 indicator- triethylamine) 22
20) Hexaisopropylamino-cyclotriphosphazene (IPPN) 22
21) Hexabenzylamino-cyclotriphosphazene (BnPN) 23
22) Hexamorpholino-cyclotriphosphazene (morphPN) 23
23) Benzoic acids without ortho groups 24
S7- Limiting Shift Measurements with Variable Background Electrolyte 25
S8- Diffusion Time Window 25
S9- Acid Mass 27
S10- References 27

S1- Data Point Selection

Although 128 data points were collected for each titration (except for morphPN which used 512 points), not all of that data can be used for pK_a determination. The top and bottom 15-20 slices are recorded at the very edge of the NMR detection coil and are thus of inferior quality and are discarded as a matter of course. Slices where the pH is such that all the indicators present are at their limiting shift values are also discarded as no useful titration data can be extracted. This results in a variable number of data points in each titration.

All indicators and analytes were assumed to be in fast exchange and were confirmed by observation of each as a single continuous sigmoidal trace in the image. Slow exchanging species display a non-continuous trace which may still be used if the two peaks can be integrated and averaged, though likely at the cost of accuracy.

S2- Niacin and Aspirin pK_a in DMSO

While the pK_a of niacin and aspirin are both unknown in neat DMSO, existing data suggests the values determined by our gradient method are likely correct. In the case of niacin, Hallé *et al.* determined the pK_a for a series of water/DMSO mixtures with increasing DMSO content.[1] The COOH dissociation exponent increases with DMSO content. At water:DMSO = 4.5:95.5, the highest DMSO content reported, a pK_a of 8.14 was found. Following the trend of this data to 100% DMSO, a pK_a of 8.54 is expected, which is in line with the value determined in this work of 8.60 ± 0.1.

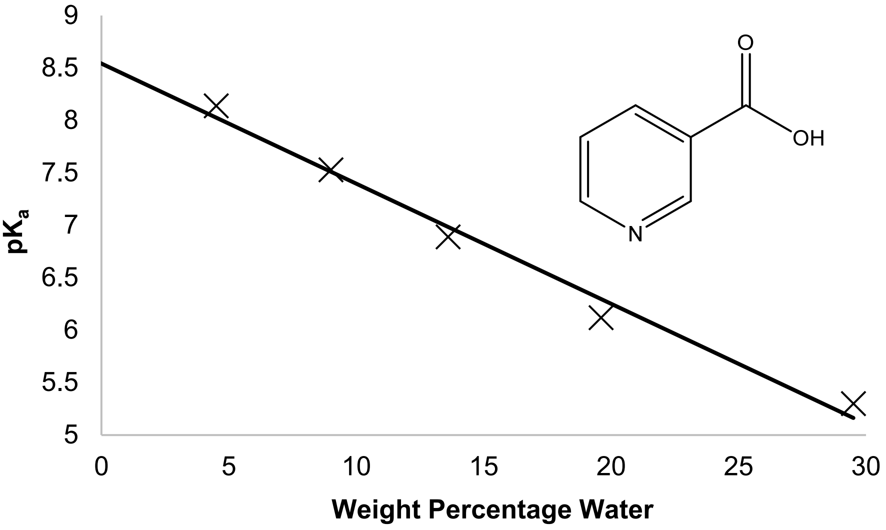

Figure S1- Niacin pK_{a1} in water-DMSO mixtures from Hallé *et al.* with an extrapolated pK_{a1} in 100% DMSO of 8.54 (R^2 = 0.99).

The most similar compound to aspirin with available pK_a data in DMSO is 2-acetamidobenzoic acid (pK_a = 8.2 ±0.1).[2] The conjugate base of 2-acetamidobenzoic acid is stabilised by an intramolecular hydrogen bond between the carboxylate and the amide proton. This makes deprotonation of the 2-acetamidobenzoic acid more favourable relative to an acid such as aspirin which does not possess a stabilising internal hydrogen bond in its conjugate base. This expectation is borne out in the determined pK_a of aspirin (8.68 ±0.1).

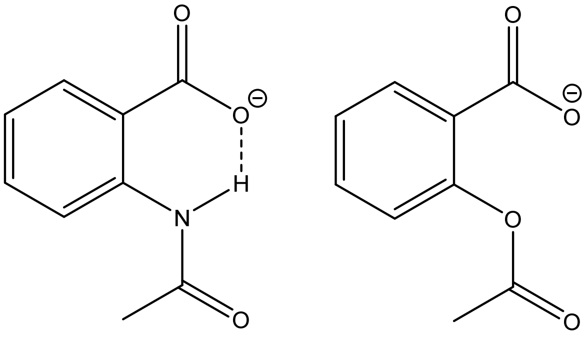

Figure S2- Intramolecular Hydrogen bonding is present in conjugate base of 2-acetamidobenzoic acid but not in asprin.

S3- Indicator Data

**Table S1. Independently measured limiting shift and literature pK_a data for basic indicators used in this work**			
Indicator	δ_L /ppm	δ_H /ppm	pK_a^{lit}
2,6 lutidine (lut)	6.9776	7.6434	4.46[3] ±0.03
1-methylimidazole (mzl)	3.5976	3.9401	6.15[4] ±0.01
imidazole (izl)	7.5909	9.0574	5.1[5] ±0.2, 6.26[4] ±0.06, 6.37[6] ±0.04, 6.94[7] ±0.06
dimethylbenzylamine (dmb)	3.3215	4.2936	7.60[8] ±0.1
morpholine (mor)	3.4481	3.7256	8.94[9] ±0.1
triethylamine (tea)	0.8871	1.1521	9.0[10] ±0.1
benzylamine (bza)	3.7025	4.1005	9.81[11] ±0.1
diethylamine (dea)	0.9431	1.1312	10.4[12] ±0.1
pyrrolidine (pyr)	1.5025	1.8134	11.06[6] ±0.04

**Table S2. Literature pKa data for acids used in this work**		
Acid	pK_a^{lit}	ref
methanesulfonic acid	1.6 ±0.1	[13]
saccharin	4.0 ±0.1	[13]
Meldrum's acid	7.32 ±0.01	[14]
barbituric acid	8.4 ±0.1	[13]
salicylic acid	6.8 ±0.1	[10]
2,4-dinitrobenzoic acid	6.52 ±0.15	[15]

S4- Automatic correction of the analyte pKa when the indicator and analyte have the same charges

The effective pK_a of a compound, $pK_{a,eff}$, is defined in terms of the true pH and the concentrations of the protonated, [HA], and non-protonated, [A], forms of the compound:

$$pK_{a,eff} = pH - \log_{10}\frac{[A]}{[HA]} \qquad S1$$

The ratio [A]/[HA] is determined from the chemical shift of the compound, as in Equation 1. $pK_{a,eff}$ is converted to the thermodynamic pK_a, $pK_{a,0}$, by including the activity coefficients, γ, of the protonated and deprotonated forms of the compound:

$$pK_{a,0} = pK_{a,eff} - \log_{10}\frac{\gamma_A}{\gamma_{HA}} \qquad S2$$

Using $pK_{a,0}$ in Equation 1, instead of $pK_{a,eff}$, yields an apparent pH, pH_{app}:

$$pH_{app} = pK_{a,0} + \log_{10}\left[\frac{\delta_{obs}-\delta_H}{\delta_L-\delta_{obs}}\right] \qquad S3$$

Combining Equations S2 and S3, pH_{app} can be related to the true pH by Equation S4:

$$pH_{app} = pH - \log_{10}\frac{\gamma_A}{\gamma_{HA}} \qquad S4$$

Assuming γ depends only on the charge of the species,[16, 17] the term γ_A/γ_{HA} will be the same for an indicator and analyte which possess the same charges as each other in their protonated and deprotonated forms. In this case, we can re-write Equation 2 in terms of pH_{app}, and $pK_{a,0}$ of the analyte:

$$\delta_{obs} = \frac{\delta_H 10^{(pK_{a,0}-pH_{app})}+\delta_L}{1+10^{(pK_{a,0}-pH_{app})}} \qquad S6$$

as

$$pK_{a,eff,analyte} - pH = pK_{a,0,analyte} + \log_{10}\frac{\gamma_{A,analyte}}{\gamma_{HA,analyte}} - \left(pH_{app} + \log_{10}\frac{\gamma_{A,indicator}}{\gamma_{HA,indicator}}\right) \qquad S7$$

Using $pK_{a,0}$ of the indicator in Equation 1 and using this apparent pH value in Equation 2 thus yields the thermodynamic pK_a of the analyte directly, provided the indicator and analyte have the same charges in their protonated and deprotonated forms.

S5- Experimental details

Experiments were performed in Norell 502 NMR tubes on a Bruker AV-I 400 or AV-II 400 spectrometer operating at 400.13 and 400.20 MHz for ^{1}H respectively. CSI experiments were carried out using the gradient phase encoding sequence of Wallace *et al*. [18] This sequence uses the WATERGATE (**W5**) pulse train of Liu *et al.* (Bruker Library ZGGPW5) to supress the solvent resonance. [19] To correspond to a 4000 Hz separation between nulls, a delay of 250 µs was used between selective pulses in the **W5** train. The pulse sequence follows **W5**-τ_1-**g**- τ_2-acquire, where τ_1 is a delay of 10 µs, **g** is the phase encoding gradient pulse and τ_2 is a delay of 200 µs. The phase encoding gradient pulse **g** was 242 µs and varied from -27 to 27 Gcm^{-1} in 128 slices. Typically, 16 dummy scans preceded signal acquisition, with 8 scans acquired for each gradient increment, with an acquisition time of 1 s. A spoil gradient of 27 Gcm^{-1} was included after the acquisition period to destroy any remaining transverse magnetisation. Time domain data files were transformed without zero-filling using sine bell apodization. A 128 slice CSI experiment had a total acquisition time of 20 minutes with a theoretical spatial resolution of 0.20 mm. For further experimental details see Wallace *et.al.* 2018.[18]

LiCl was dried under vacuum at 100°C for 3 hours and stored in a sealed flask in a nitrogen purged glovebox. Phosphazenes were prepared according to literature methods.[20] Anhydrous DMSO, LiCl and 2,6 lutidine were purchased from Sigma Aldrich. All other materials were purchased from Alfa Aesar and used without further purification.

All solutions were prepared with anhydrous DMSO as 5 ml stock solutions in a N_2 purged glovebox. Volatile reagents deleterious to the glovebox were added in air, with no observable change in the water content or in the determined pK_a of the resulting solutions. (See results section and Table S9 for more information on water concentration). All stock solutions were prepared with a 20 mmol concentration of hexamethyldisilane to act as a pH independent internal chemical shift reference.

To establish a pH gradient, solid acid was weighed directly into the NMR tube using a Mettler AE101 balance with a stated precision of ± 0.01 mg. Variance in the amount of acid added over the range 2 to 10 mg was found to have no effect on the precision of pK_a^{det}, provided the mass of acid is in excess over the total base concentration of the DMSO solution (ESI S8). Wallace *et al.* have previously shown that there is a wide acceptable range of gradient strengths that yield accurate and reproducible titration data in water.[18] We have found that, in DMSO, an acid excess greater than five times over the total base concentration results in a very sharp pH gradient and consequently poorer spatial definition of the titration data.

Acidic analytes were used as the diffusing acid (Table 1) and titrated against two basic indicators (method A). Four 2 mm glass beads were placed on top of the solid acid in the NMR tube to prevent rapid mixing. 550 µL of basic solution was then gently layered on top of the glass beads and the tube left to stand vertically in a 25°C water bath until analysis. Basic analytes were investigated by titrating a solid acid (Table S2) of known pK_a against a solution containing the analyte and two basic indicators (method B).

The time required to establish the pH gradient is longer in DMSO compared with aqueous solution due to slower diffusion in the more viscous solvent affording a very wide time window in which an acceptable pH gradient and useful data can be obtained. Data obtained between 8-32 hours after

sample preparation yielded acceptable gradients and accurate pK_a data, with data obtained after 16-24 hours containing the highest number of useable data points (S7). Samples left at ambient laboratory temperature, 20°C, as opposed to measurement temperature, 25°C, took considerably longer to establish optimal gradients.

Limiting shifts of indicators were determined independently from the imaging experiments. An excess of strong acid or base was used to measure limiting shifts of all reagents, except for phosphazenes for which weaker acids close to pK_{a1} of the phosphazenes were used since multiple protonation of the ring nitrogen sites is possible.

The tracked resonances of analytes and indicators were selected on the basis of sensitivity to pH and presence in an uncrowded spectral region to minimize overlap with other resonances.

Table S3. Tracked resonances of indicators and analytes in this work

Compound	δ_L /ppm	δ_H /ppm	Henderson-Hasselbalch plot
2,6 lutidine	6.978	7.643	-
1-methylimidazole	3.598	3.940	S6-4
imidazole	7.591	9.057	S6-1
dimethylbenzylamine	3.322	4.294	-
morpholine	3.448	3.726	S6-7
triethylamine	0.887	1.152	-
benzylamine	3.703	4.101	S6-10
diethylamine	0.943	1.131	S6-13
pyrrolidine	1.503	1.813	-
2,4-dinitrobenzoic acid	8.429	8.745	S6-14
salicylic acid	7.605	7.751	S6-15, 16
aspirin	7.432	7.588	S6-17
niacin	8.496	8.694	S6-18, 19
benzoic acid	7.813	8.001	-
m-toluic acid	7.693	7.802	-
p-chlorobenzoic acid	7.806	8.027	-
Hexaisopropylamino-cyclotriphosphazene	1.003	1.057	S6-20
Hexabenzylamino-cyclotriphosphazene	7.168	7.221	S6-21
Hexamorpholino-cyclotriphosphazene	3.475	3.542	S6-22

**Table S4. Experimental details for indicator titrations in DMSO solution**							
Indicator	Literature pK_a	Determined pK_a ± 0.1 (**average**)	Mass of acid /mg (**B**/**M**/**S**)[a]	Ionic Strength /M	δ_H from fit	δ_L from fit	Titration components /mM
1-methylimidazole	6.15 ± 0.01	6.19	4.3 **S**	0.01	3.825	3.601	10 **lut**, 10 **mzl**, 10 **izl**
		6.15	5.0 **S**	0.01	3.826	3.603	15 **lut**, 1 **mzl**, 15 **izl**
		6.16 (**6.16**)	3.5 **S**	0.01	3.827	3.603	15 **lut**, 0.1 **mzl**, 15 **izl**
imidazole	5.1 ± 0.2, 6.26 ± 0.06, 6.37 ± 0.04, 6.94 ± 0.06	6.46, 6.45 (**6.46**)	9, 4.7 **S**	0.02, 0.02	9.548, 9.592	7.567, 7.599	10 **mzl**, 15 **izl** 10 **dmb**
morpholine	8.94 ± 0.1	9.00, 9.01 (**9.01**)	5.0, 5.0 **M**	0.02, 0.02	3.723, 3.722	3.406, 3.405	10 **dmb**, 10 **morph**, 10 **tea**
benzylamine	9.81 ± 0.1	9.78, 9.78 (**9.78**)	5.0, 5.3 **B**	0.02, 0.02	4.208, 4.210	3.663, 3.664	10 **bzl**, 10 **dea**, 10 **pld**
diethylamine	10.4 ± 0.1	10.41, 10.43 (**10.42**)	5.3, 4.7 **B**	0.02, 0.02	1.159, 1.131	0.988, 0.997	10 **bzl**, 10 **dea**, 10 **pld**

[a] Acid diffusant **S** = saccharin, **M** = Meldrum's acid, **B** = barbituric acid

Table S5. Imidazole titrations with different masses of saccharin						
Mass of saccharin /mg	Determined pKa ± 0.1	ΔpK_a from determined value	Ionic Strength /M	Acidic LS from fit	Basic LS from fit	Titration components /mM
2	6.46	0	0.02	9.617	7.547	10 **mzl**, 15 **izl**, 10 **dmb**
4	6.46	0	0.02	9.596	7.555	
6	6.46	0	0.02	9.586	7.553	
8	6.50	+0.04	0.02	9.565	7.501	
10	6.47	+0.01	0.02	9.555	7.563	

Table S6. Time resolved imidazole titration against 4.2 mg of saccharin						
Diffusion time /hrs	Determined pK_a ± 0.1	ΔpK_a from reported value	Ionic Strength /M	Acidic LS from fit	Basic LS from fit	Titration components /mM
4	N/A (no gradient visible)	N/A	N/A	N/A	N/A	10 **mzl**, 15 **izl**, 10 **dmb**
8	6.44	-0.02	0.02	9.626	7.556	
12	6.45	-0.01	0.02	9.599	7.568	
16	6.47	+0.01	0.02	9.572	7.550	
20	6.46	0	0.02	9.601	7.548	
24	6.47	+0.01	0.02	9.583	7.543	
28	6.48	+0.02	0.02	9.573	7.531	
32	6.49	+0.03	0.02	9.552	7.523	

Table S7. Experimental details for titrations with acidic analytes

Analyte	Literature pK_a	Determined pK_a (**average**)	Mass of acid /mg	Ionic Strength /M	Acidic LS from fit	basic LS from fit	Titration components
2,4-dinitrobenzoic acid	6.52 ± 0.15	6.54, 6.48 (**6.51**)	4.8, 5.0	0.02, 0.02	8.706, 8.784	8.439, 8.418	15 **mzl**, 15 **dmb**
salicylic acid	6.8 ± 0.1	6.80	4.8	0.01	7.750	7.597	20 **mzl**, 20 **izl**
		6.77 (**6.78**)	5.9	0.02	7.752	7.612	15 **izl**, 15 **dmb**
aspirin	-	8.64, 8.72 (**8.68**)	5.5, 5.7	0.02, 0.01	7.589, 7.587	7.449, 7.415	15 **dmb**, 15 **tea**
niacin	-	8.60	5.3	0.02	8.718	8.522	15 **dmb**, 15 **tea**
		8.59 (**8.60**)	5.0	0.02	8.670	8.470	15 **tea**, 15 **bzl**
benzoic acid	11.0 ± 0.3	9.29, 9.36 (**9.33**)	7.4, 8.6	0.03, 0.03	8.006, 7.996	7.814, 7.812	10 **bzl**, 10 **dea**, 15 **pld**
m-toluic acid	11.0 ± 0.3	8.80	5.5	0.03	7.802	7.693	10 **bzl**, 10 **dea**, 15 **pld**
p-chlorobenzoic acid	10.1 ± 0.3	9.02	5.8	0.03	8.027	7.806	10 **morph**, 10 **tea**, 20 **bzl**

**Table S8. Experimental details for titrations with phosphazenes**						
Analyte	Determined pK_a ± 0.1	Mass of acid /mg (**S/B**)[a]	Ionic Strength /M	Acidic LS from fit	Basic LS from fit	Titration components /mM
**IPPN**	11.65	6.0 **B**	0.01	1.057	1.003	15 **DEA**, 15 **PLD**, 5 **IPPN**
**BNPN**	9.80	2.4 **B**	0.01	7.221	7.168	10 **morph**, 10 **dea**, 10 **BNPN**
**MLPN**	4.22	7.6 **S**	0.02	3.542	3.475	10 **lut**, 10 **izl**, 2 **MLPN**

[a] Acid diffusant used **S**: saccharin, **B** : barbituric acid

**Table S9. Experimental details for titrations with background LiCl electrolyte**							
Indicator	Literature pK_a	Determined pK_a (**average**) (**S/M**)[a]	Mass of acid /mg	Ionic Strength /M	Acidic LS from fit	basic LS from fit	Titration components
1-methylimidazole	6.15 ± 0.01	6.17 **S**	4.0	0.11	3.819	3.602	10 **lut**, 10 **mzl**, 15 **izl**
		6.14 **S**	4.0	0.21	3.815	3.604	
morpholine	8.94 ± 0.1	9.03 **M**	5.0	0.13	3.780	3.378	10 **dmb**, 10 **morph**, 10 **tea**
		8.97 **M**	5.0	0.22	3.802	3.423	

[a] Acid diffusant **S**: saccharin,**M** Meldrum's acid

Table S10. Experimental details for titrations with water doped DMSO								
Indicator	Reported pK_a ± 0.1	Water percentage	Determined pK_a ± 0.1	Mass of acid /mg (**S**/**B**)[a]	Ionic Strength /M	Acidic LS from fit	Basic LS from fit	Titration components /mM
imidazole	6.46	1	6.40	4.3 **S**	0.02	9.650	7.571	10 **mzl**, 15 **izl**, 10 **dmb**
		2	6.44	8.1 **S**	0.02	9.588	7.528	
benzylamine	9.79	1	9.80	5.0 **B**	0.05	4.170	3.662	20 **bzl**, 20 **dea**, 20 **pld**
		2	9.79	5.0 **B**	0.05	4.145	3.661	

[a] Acid diffusant used **S**: saccharin, **B** : barbituric acid

S6- Titration Data

The ionic strength at the titration midpoint is determined by extracting the percentage ionisation for the indicators and analyte from the chemical shifts at the slice where pH most closely matches pK_a and converting to ionic strength. The ionic strength at the midpoint of the titration is used as a representative ionic strength in this work as it is at this pH that the fitted pK_a is most sensitive to ionic strength. The error in the tabulated ionic strength values is derived from the error in the measured chemical shifts, with a maximum error of ±0.0003 M.

1) Imidazole (anhydrous)

4.3 mg Saccharin
Midpoint ionic strength 0.02 M
pKa = 6.46 ± 0.1
56 data points

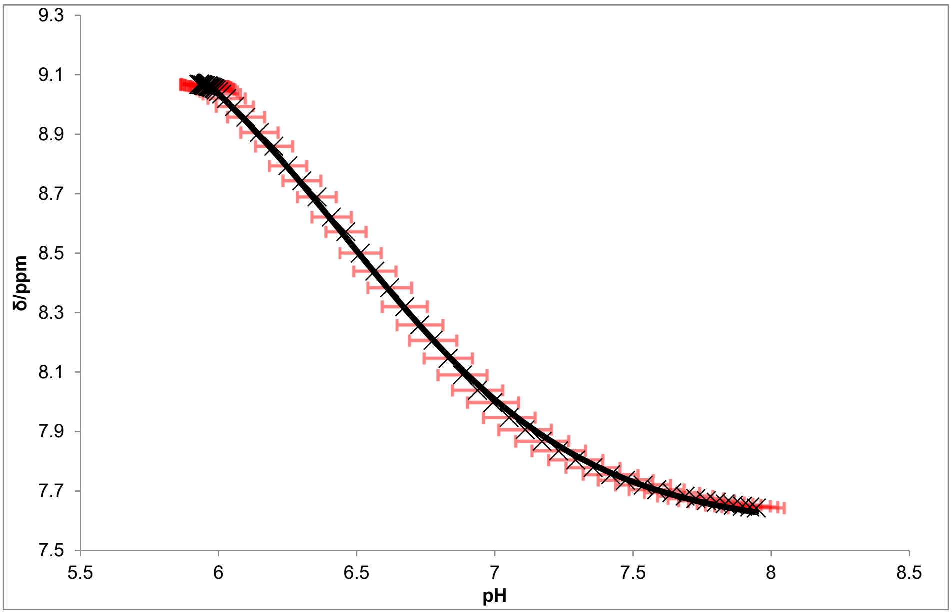

2) Imidazole 1% water

4.3 mg Saccharin
Midpoint ionic strength 0.02 M
pK_a = 6.40 ± 0.1
58 data points

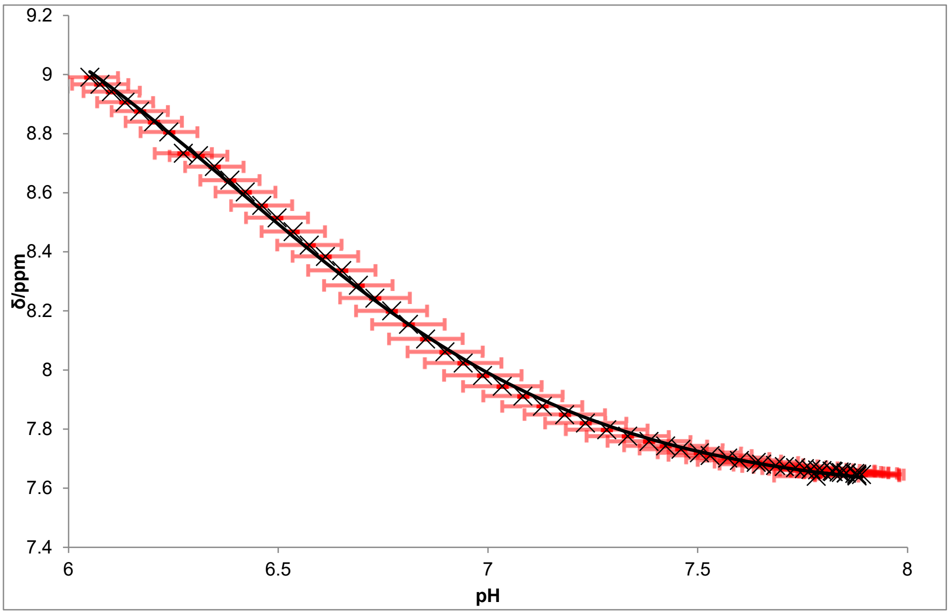

3) Imidazole 2% water

8.1 mg Saccharin
Midpoint ionic strength 0.02 M
pK_a = 6.44 ± 0.1
58 data points

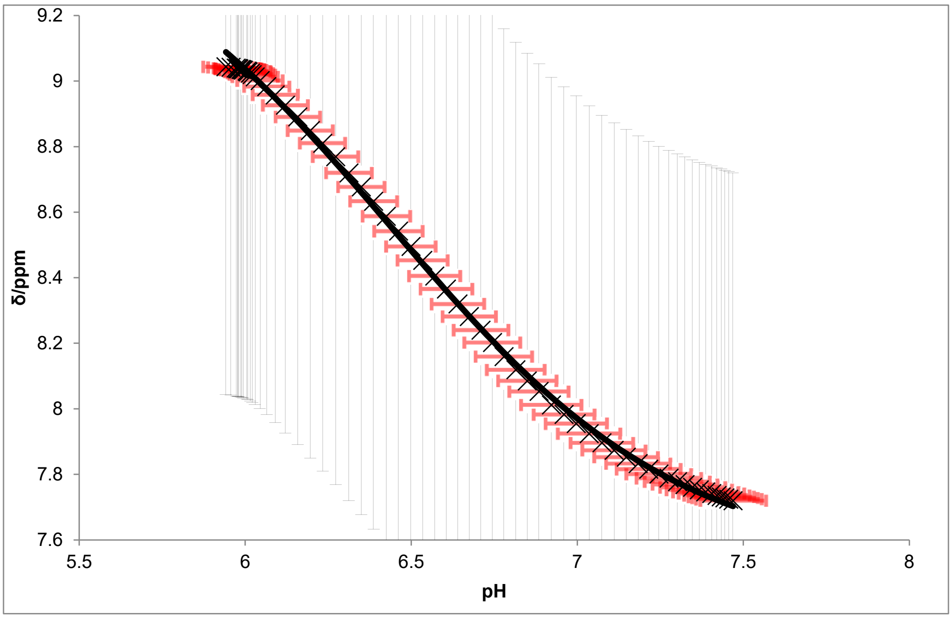

4) 1-methylimidazole

5.0 mg saccharin
Midpoint ionic strength 0.01 M
pK_a = 6.15 ± 0.1
53 data points

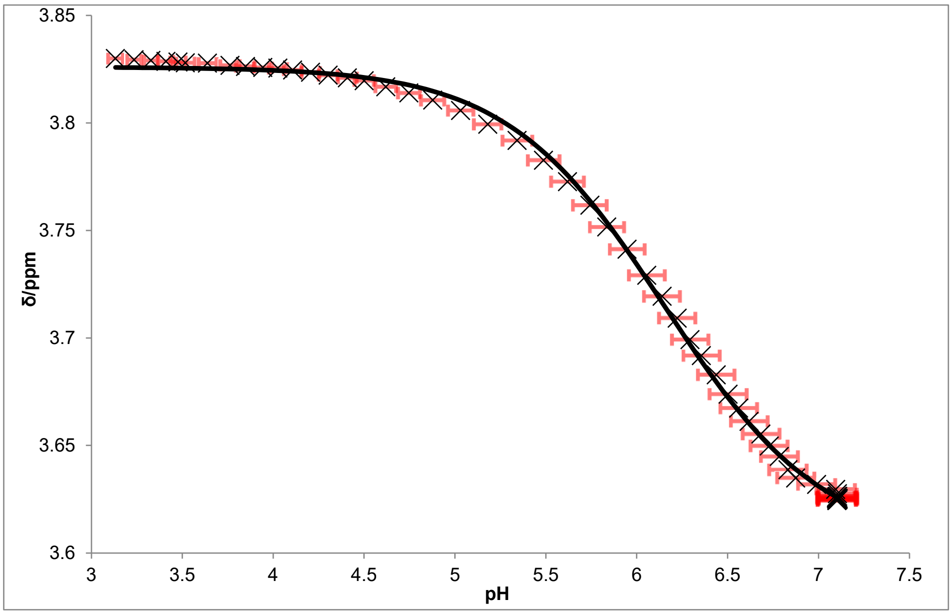

5) **1-methylimidazole 0.1M LiCl background electrolyte**

4.0 mg saccharin
Midpoint ionic strength 0.11 M
pKa = 6.14 ± 0.1
48 data points

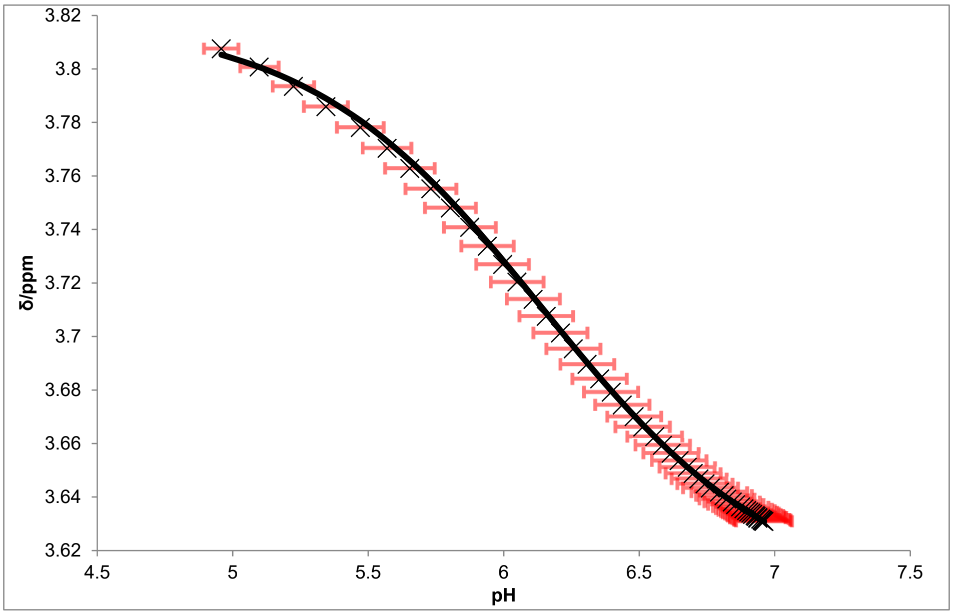

6) **1-methylimidazole 0.2M LiCl background electrolyte**

4.0 mg saccharin
Midpoint ionic strength 0.21 M
pKa = 6.14 ± 0.1
33 data points

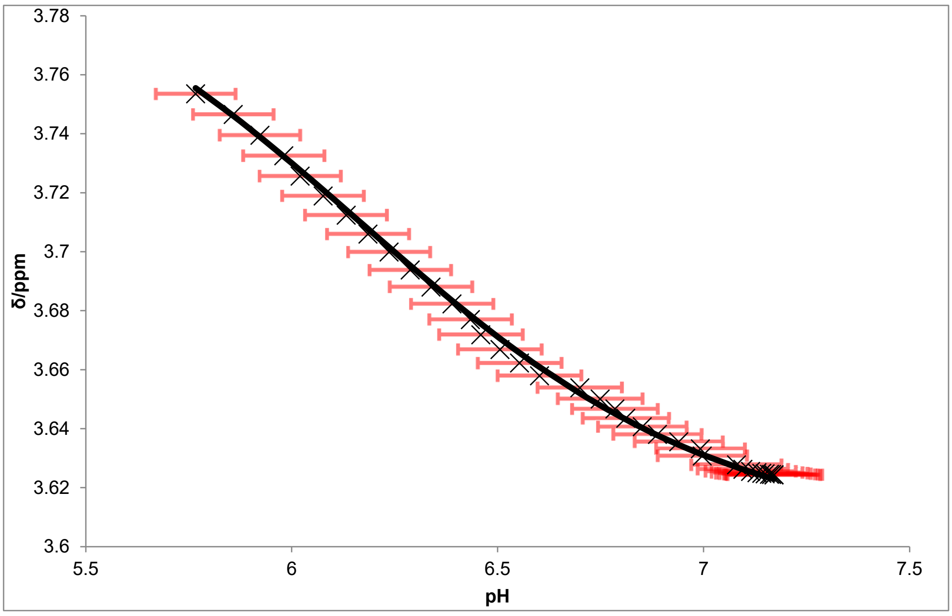

7) Morpholine

5.0 mg barbituric acid
Midpoint ionic strength 0.02 M
pK_a = 9.01 ± 0.1
30 data points

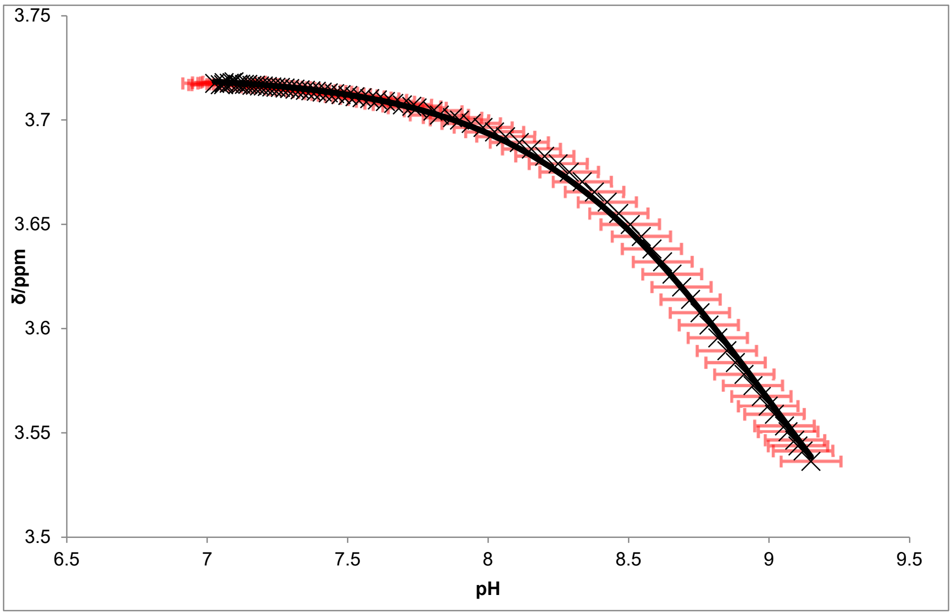

8) Morpholine 0.1M LiCl background electrolyte

5.0 mg barbituric acid
Midpoint ionic strength 0.13 M
pKa = 9.03 ± 0.1
21 data points

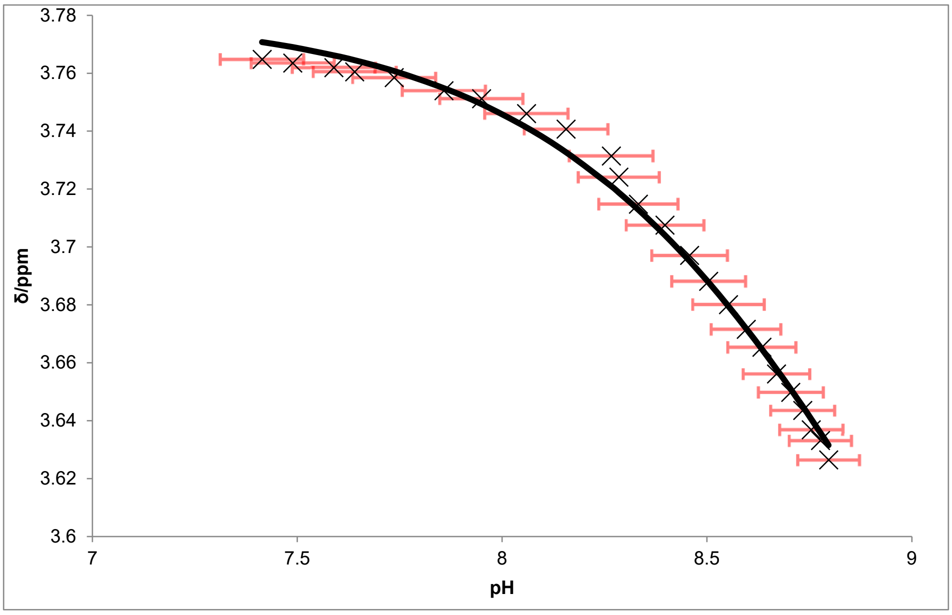

9) Morpholine 0.2M LiCl background electrolyte

5.0 mg barbituric acid
Midpoint ionic strength 0.22 M
pKa = 8.97 ± 0.1
27 data points

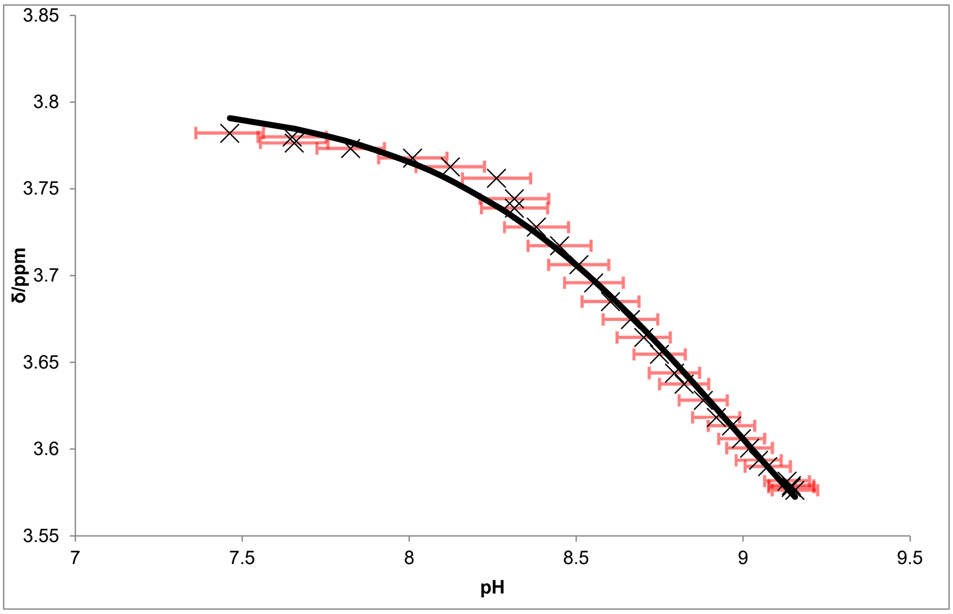

10) Benzylamine (Anhydrous)

5.0 mg barbituric acid
Midpoint ionic strength 0.05 M
pKa = 9.78 ± 0.1
66 data points

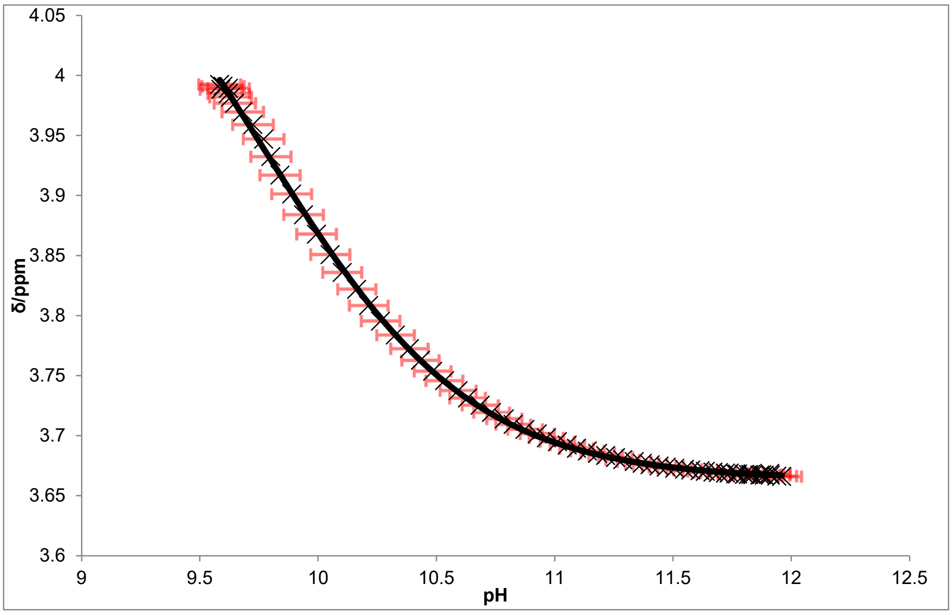

11) Benzylamine 1% Water

5.0 mg barbituric acid
Midpoint ionic strength 0.05 M
pKa = 9.80 ± 0.1
48 data points

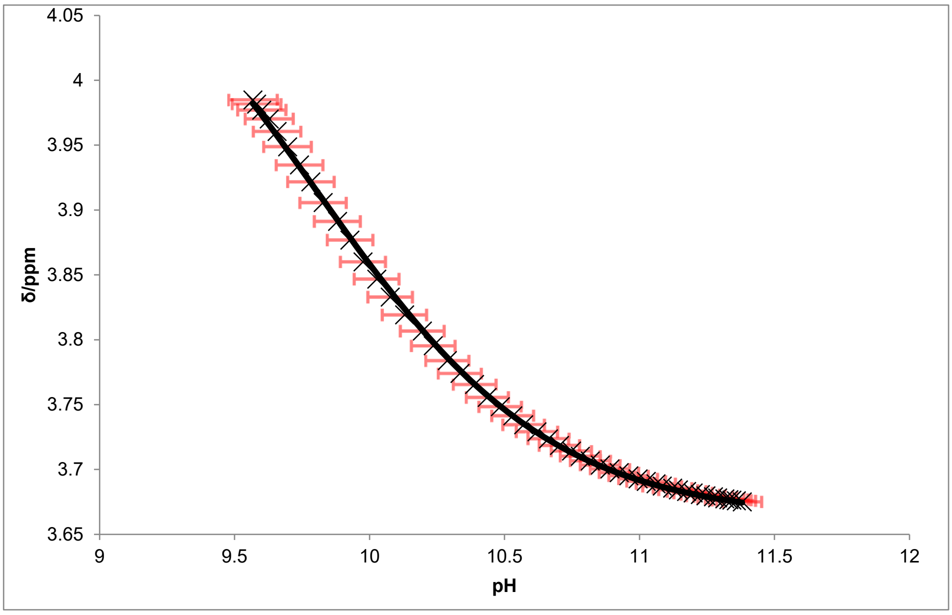

12) Benzylamine 2% Water

5.0 mg barbituric acid
Midpoint ionic strength 0.05 M
pKa = 9.79 ± 0.1
51 data points

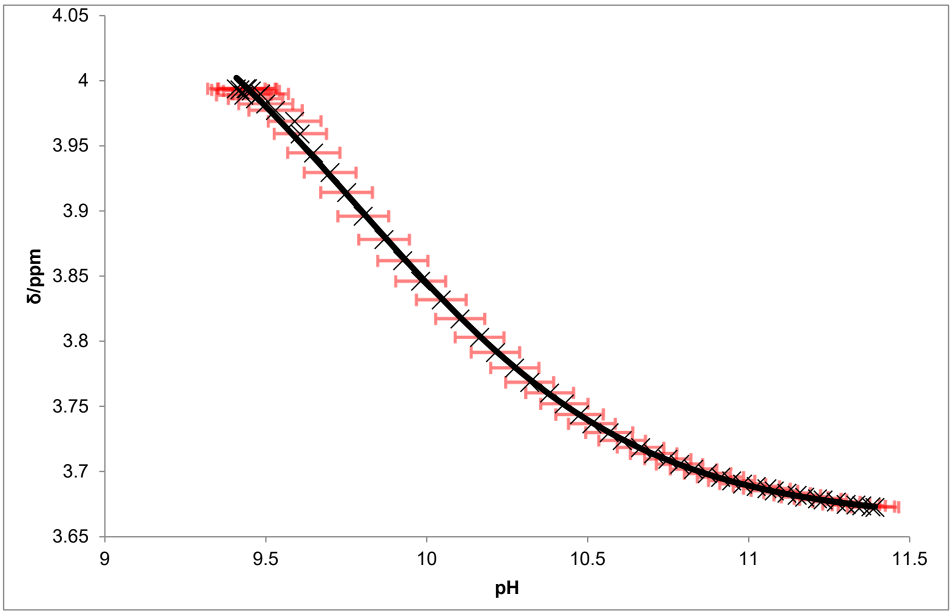

13) Diethylamine

5.3 mg barbituric acid
Midpoint ionic strength 0.02 M
pKa = 10.41 ± 0.1
42 data points

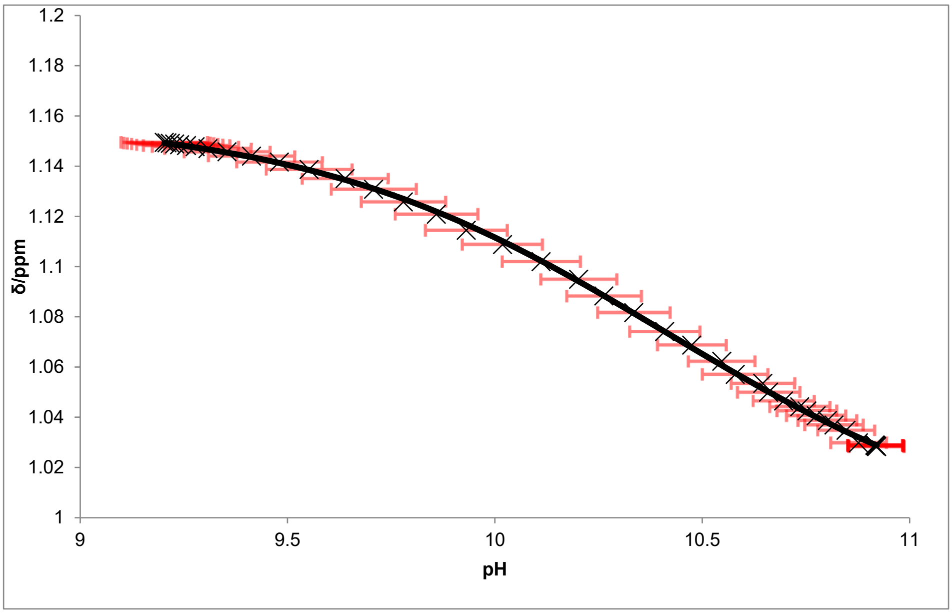

14) 2,4-dinitrobenzoic acid

4.8 mg 2,4-dinitrobenzoic acid
Midpoint ionic strength 0.02 M
pKa = 6.54 ± 0.1
48 data points

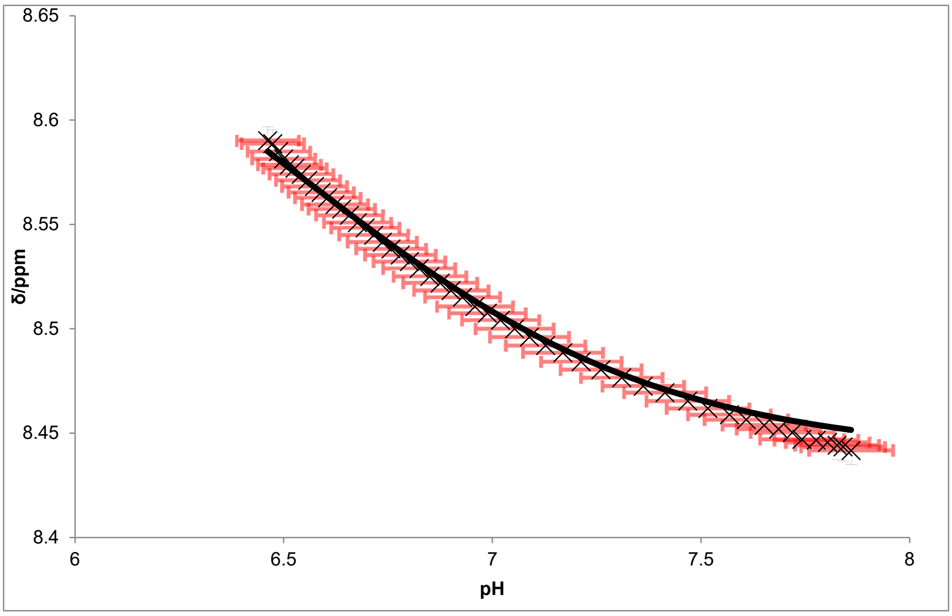

15) Salicylic acid (1-methylimidazole and imidazole)

4.8 mg salicylic acid
Midpoint ionic strength 0.01 M
pKa = 6.80 ± 0.1
35 data points

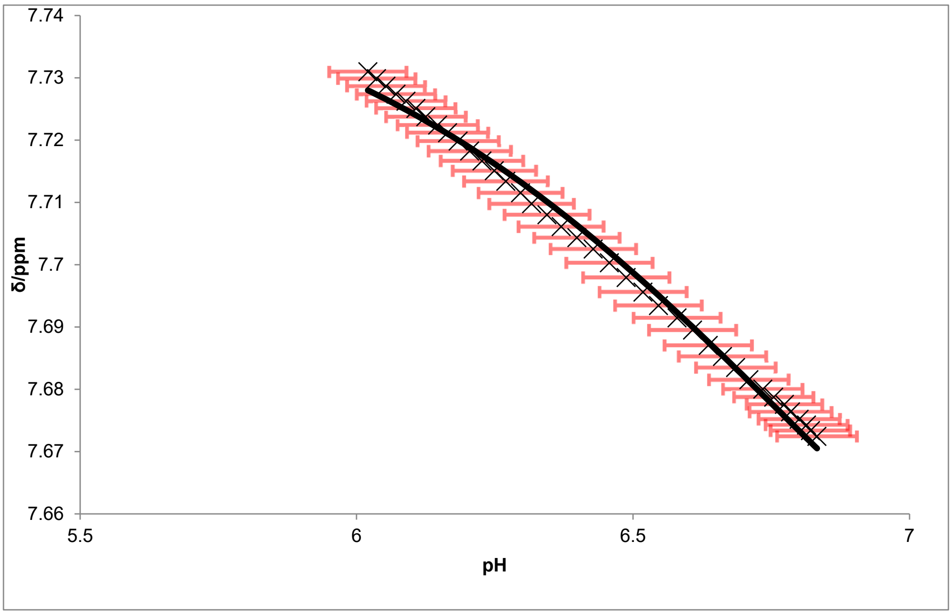

16) Salicylic acid (imidazole and dimethylbenzylamine)

5.9 mg salicylic acid
Midpoint ionic strength 0.02 M
pKa = 6.77 ± 0.1
45 data points

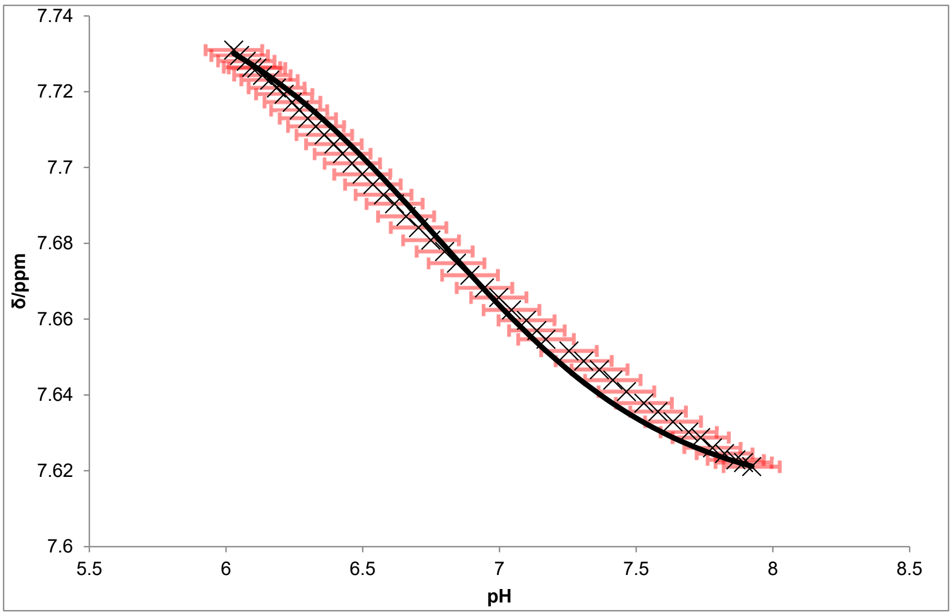

17) Aspirin

5.7 mg aspirin
Midpoint ionic strength 0.01 M
pKa = 8.72 ± 0.1
37 data points

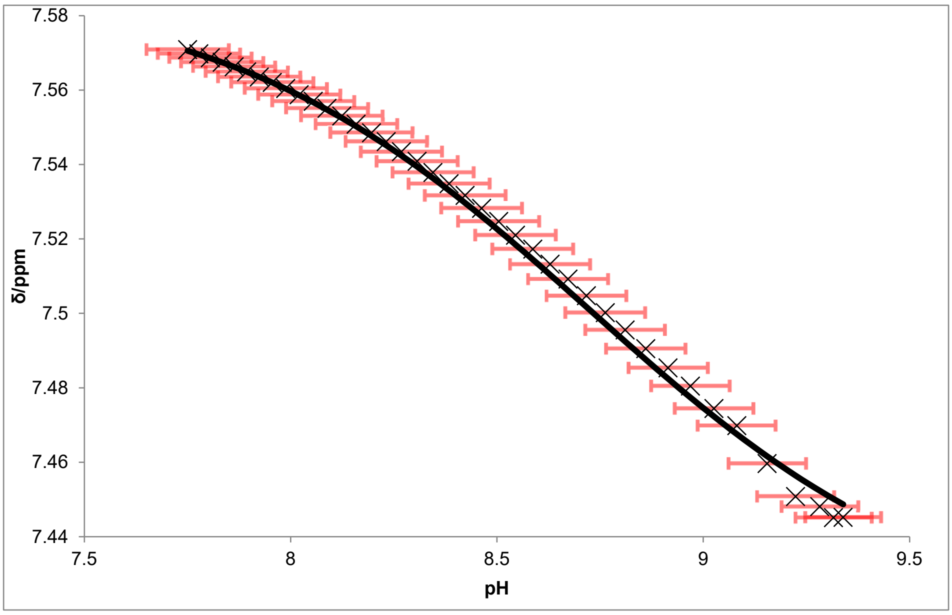

18) Niacin (2 indicators- dimethylbenzylamine and triethylamine)

5.3 mg niacin
Midpoint ionic strength 0.02 M
pKa = 8.60 ± 0.1
48 data points

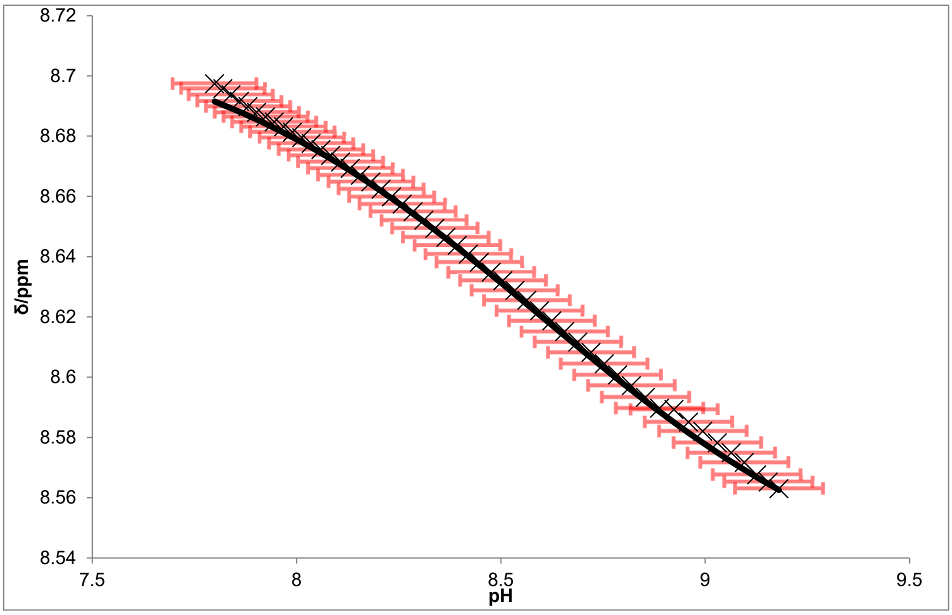

19) Niacin (1 indicator- triethylamine)

5.0 mg niacin
Midpoint ionic strength 0.02 M
pKa = 8.59 ± 0.1
52 data points

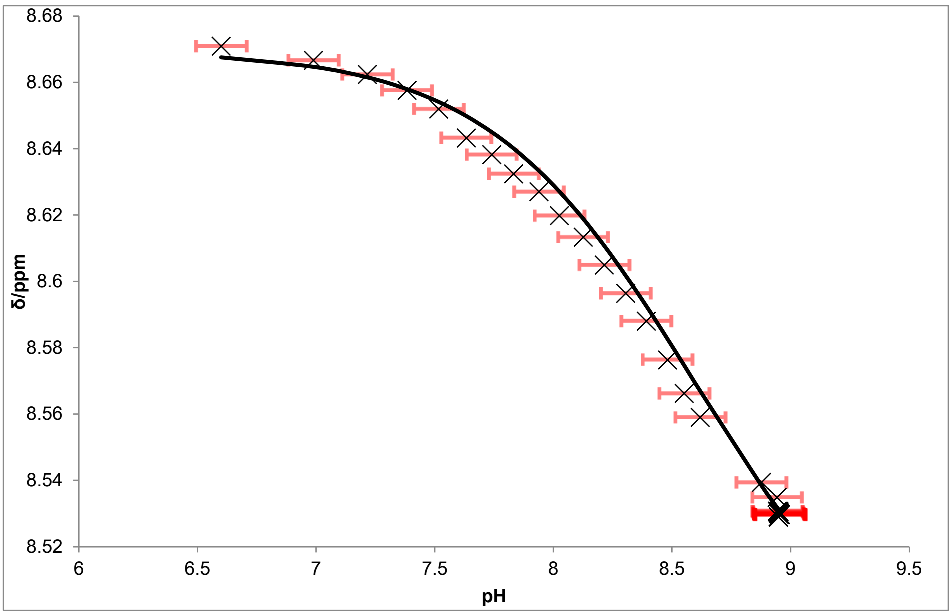

20) Hexaisopropylamino-cyclotriphosphazene (IPPN)

6.0 mg barbituric acid
Midpoint ionic strength 0.01 M
pKa = 11.65 ± 0.1
39 data points

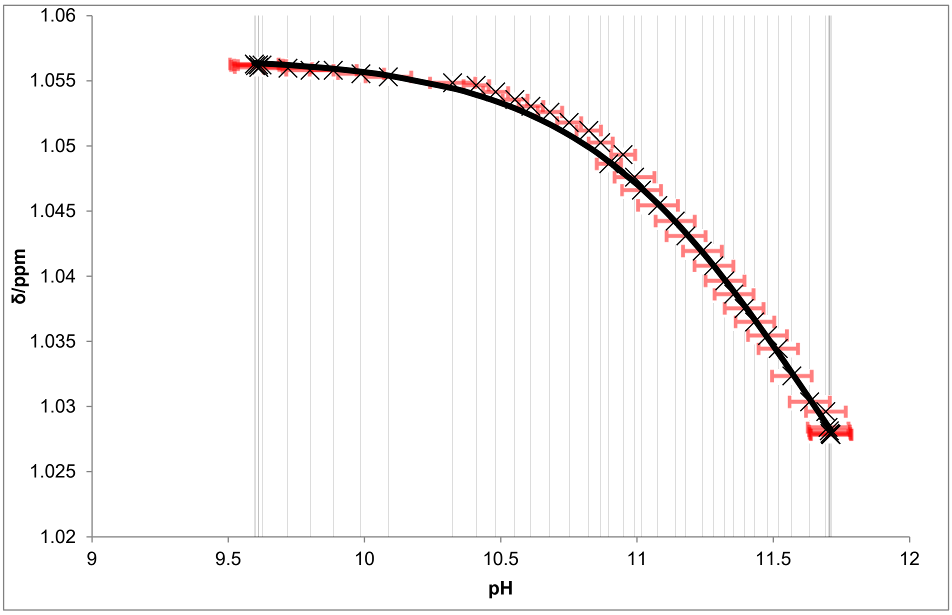

21) Hexabenzylamino-cyclotriphosphazene (BnPN)

2.4 mg barbituric acid
Midpoint ionic strength 0.01 M
pKa = 9.80 ± 0.1
38 data points

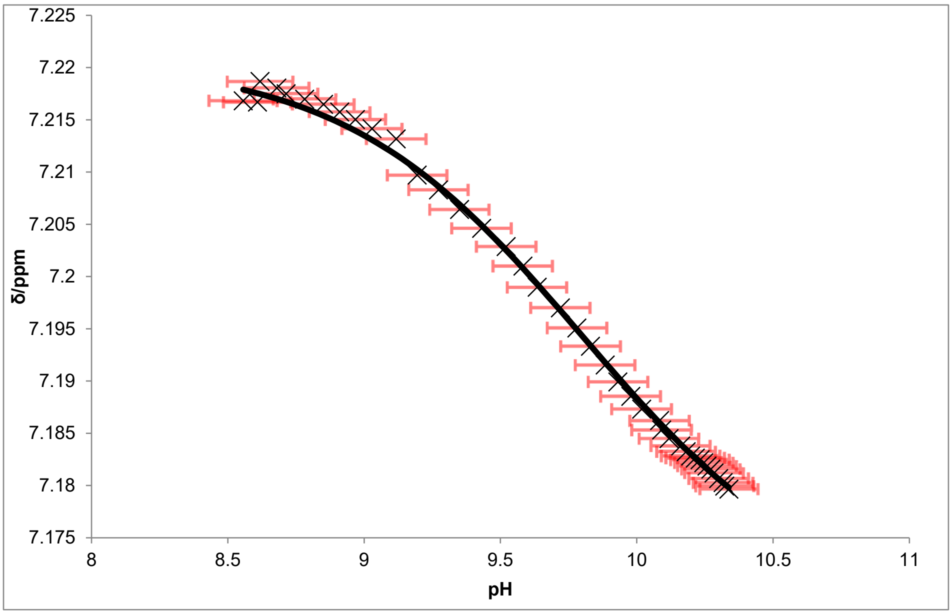

22) Hexamorpholino-cyclotriphosphazene (morphPN)

7.6 mg saccharin
Midpoint ionic strength 0.02 M
pKa = 4.22 ± 0.1
258 data points

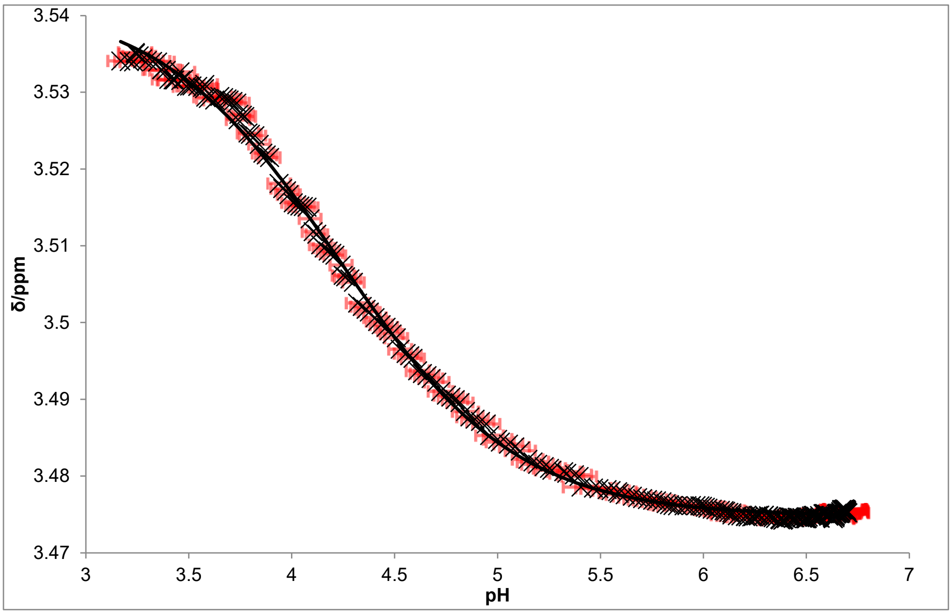

23) Benzoic acids without ortho groups

Titrations were also performed for benzoic acid, m-toluic acid and p-chlorobenzoic acid. The determined pK_a values were lower than the reported literature values due to homoassociation of the acid with its conjugate base.[21] Homoassociation is observed in acids with an isolated charge in its conjugate base (meta/para substitued benzoic acids and phenols).[22] This effect is common to all methods of titration for compounds of this type and must be corrected for. The deviation of our data from literature values (corrected to infinite dilution) is roughly the same size as a representative homoassociation constant.[23]

S7- Limiting Shift Measurements with Variable Background Electrolyte

**Table S11. Limiting shift measurements with variable background electrolyte**			
Indicator	Electrolyte Concentration	δ_L (ppm)	δ_H (ppm)
2,6 Lutidine	0 M LiCl	(3,5 CH) 6.9783	(3,5 CH) 7.7051
2,6 Lutidine	0.1 M LiCl	(3,5 CH) 6.9810	(3,5 CH) 7.6881
2,6 Lutidine	0.2 M LiCl	(3,5 CH) 6.9843	(3,5 CH) 7.6981
dimethylbenzylamine	0 M LiCl	(CH_2) 3.3257	(CH_2) 4.2554
dimethylbenzylamine	0.1 M LiCl	(CH_2) 3.3274	(CH_2) 4.2367
dimethylbenzylamine	0.2 M LiCl	(CH_2) 3.3297	(CH_2) 4.2500
triethylamine	0 M LiCl	(CH_3) 0.8871	(CH_3) 1.1521
triethylamine	0.1 M LiCl	(CH_3) 0.8884	(CH_3) 1.1660
triethylamine	0.2 M LiCl	(CH_3) 0.8892	(CH_3) 1.1689

S8- Diffusion Time Window

To illustrate the wide window of time over which a pH gradient can be used to determine pK_a accurately, 4.2 mg of saccharin was allowed to diffuse into a solution of 10 mmol 1-methylimidazole, 15 mmol imidazole and 10 mmol dimethylbenzylamine and images recorded at 4-hour intervals for 32 hours after diffusion had begun. The pK_a of imidazole was determined from each image and is given below.

**Table S12. Time resolved pK_a determination of imidazole**		
Time after diffusion start (h)	Determined pK_a ± 0.1	ΔpK_a from reported value
4	N/A (no gradient visible)	N/A
8	6.44	-0.02
12	6.45	-0.01
16	6.47	+0.01
20	6.46	0
24	6.47	+0.01
28	6.48	+0.02
32	6.49	+0.03

Data collected between 12-24 hours is of superior quality, within ±0.01 of our reported value. There is a 24-hour window in which pK_a can be determined, highlighting the flexibility of the method with respect to instrument access.

Assuming a high solubility for the acidic diffusant, the concentration of the acid, [acid], can be expected to follow a Gaussian profile with time, t (Equation S8): [18]

$$[\mathrm{Acid}] = \frac{m}{\pi r^2 M_r \sqrt{\pi D_{Ac} t}} \exp\left[\frac{-(Z-h)^2}{4 D_{Ac} t}\right] \qquad \text{S8}$$

where m is the mass of acid weighed out, M_r the molecular mass of the acid, r the tube radius (2.1 mm), h the height of the solid acid (2 mm), Z the distance from the tube base and D_{Ac} the diffusion coefficient of the acid. Based on diffusion coefficients in DMSO reported in the literature,[24,25] D_{Ac} in our experiments can be assumed to be between 2 x 10^{-10} m^2s^{-1} and 5 x 10^{-10} m^2s^{-1} for all our acidic diffusants. For the experiment described above (Table S12), the optimum time window of 12-24 hours can readily be understood by plotting the concentration of saccharin calculated at different times using Equation S8 (Figure S23). When t < 12 hours, the acid has not diffused far enough up the tube for the analyte to be fully protonated in any part of the NMR active window of the sample (ca. 11 – 25 mm from the base of the tube). A full titration curve would not be observed. When t > 24 hours, the diffusing acid has reached the upper region of the NMR window so that it would not be possible to analyse the analyte in its almost fully deprotonated state; a full titration curve would not be observed.

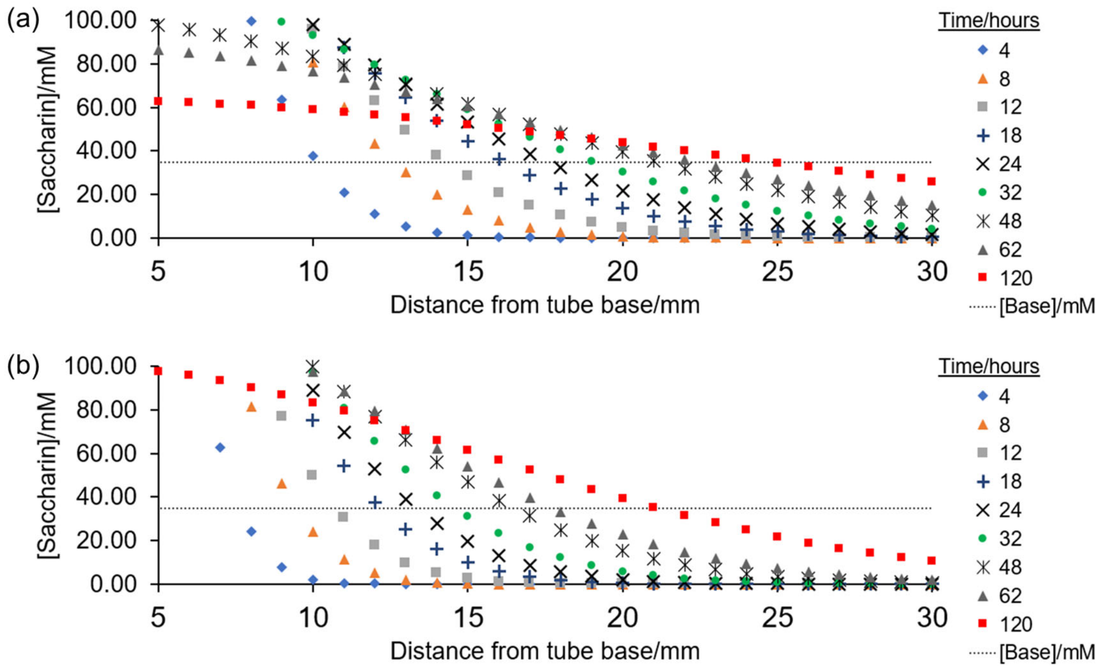

Figure S23- Plot of concentration of saccharin against distance from the base of the NMR tube, calculated using Equation S8 with D_{Ac} = 5 x 10^{-10} m^2s^{-1} (a) and 2 x 10^{-10} m^2s^{-1} (b). The NMR-active region of our NMR probe stretches ca. 7 mm either side of the tube centre (ca. 18 mm). m = 4.2 mg, M_r = 183.18 g/mol.

S9- Acid Mass

To demonstrate the broad range of acceptable pH gradients, the pK_a of imidazole was determined using a series of samples with variable diffusing acid masses (2-10 mg) recorded 20-24 hours after the start of diffusion. These results are in close agreement with each other and our reported value.

Table S13. Repeat titrations of imidazole with varying mass of saccharin

Acid Mass (mg)	Determined pK_a ± 0.1	ΔpK_a from reported value
2	6.46	0
4	6.46	0
6	6.46	0
8	6.50	+0.04
10	6.47	+0.01

S10- References

1. Hallé, J. C.; Lelievre, J.; Terrier, F., Solvent effect on preferred protonation sites in nicotinate and isonicotinate anions. *Can. J. Chem.* **1996,** *74* (4), 613-620.
2. Emenike, B. U.; Liu, A. T.; Naveo, E. P.; Roberts, J. D., Substituent Effects on Energetics of Peptide-Carboxylate Hydrogen Bonds as Studied by 1H NMR Spectroscopy: Implications for Enzyme Catalysis. *J. Org. Chem.* **2013,** *78* (23), 11765-11771.
3. Benoit, R. L.; Fréchette, M.; Lefebvre, D., 2,6-Di-tert-butylpyridine: an unusually weak base in dimethylsulfoxide. *Can. J. Chem.* **1988,** *66* (5), 1159-1162.
4. Benoit, R. L.; Boulet, D.; Séguin, L.; Fréchette, M., Protonation of purines and related compounds in dimethylsulfoxide and water. *Can. J. Chem.* **1985,** *63* (6), 1228-1232.
5. Neuvonen, H.; Neuvonen, K., Investigation of the applicability of cis-urocanic acid as a model for the catalytic Asp–His dyad in the active site of serine proteases based on 1H NMR hydrogen bonding studies and spectroscopic pKa measurements. *J. Chem. Soc., Perkin Trans. 2* **1998,** (7), 1665-1670.
6. R. Crampton, M.; A. Robotham, I., Acidities of Some Substituted Ammonium Ions in Dimethyl Sulfoxide†. *J. Chem. Res., Synop* . **1997,** (1), 22-23.
7. Kozak, A.; Czaja, M.; Chmurzyński, L., Investigations of (acid+base) equilibria in systems modelling interactions occurring in biomolecules. *J. Chem. Thermodyn.* **2006,** *38* (5), 599-605.
8. Ritchie, C. D.; Lu, S., Proton transfers among oxygen and nitrogen acids and bases in dimethyl sulfoxide solution. *J. Amer. Chem. Soc.* **1990,** *112* (21), 7748-7756.
9. Um, I.-H.; Lee, E.-J.; Jeon, S.-E., Effect of solvent on reactivity and basicity: aminolyses of p-nitrophenyl acetate in H2O and in DMSO. *J. Phys. Org. Chem.* **2002,** *15* (8), 561-565.
10. Kolthoff, I. M.; Chantooni, M. K.; Bhowmik, S., Dissociation constants of uncharged and monovalent cation acids in dimethyl sulfoxide. *J. Amer. Chem. Soc.* **1968,** *90* (1), 23-28.
11. Bowden, K.; Hirani, S. I. J., The acidity of weak carbon acids. Part 5. The kinetic acidities of substituted benzyl cyanides using substituted benzylamines as bases. *J. Chem. Soc., Perkin Trans. 2* **1990,** (11), 1889-1891.
12. Bowden, K.; Nadvi, N., The Acidity of Weak Carbon Acids. Part 3. The Kitetic Activities of Substituted Benzyl Cyanides using Secondary Aliphatic Amines and Guanidines as Bases. *J. Chem. Res., Miniprint* **1990,** (10), 2473-2481.
13. Taft, R. W.; Bordwell, F. G., Structural and solvent effects evaluated from acidities measured in dimethyl sulfoxide and in the gas phase. *Acc. Chem. Res.* **1988,** *21* (12), 463-469.
14. Arnett, E. M.; Harrelson, J. A., Ion Pairing and Reactivity of Enolate Anions. 7. A Spectacular Example of the Importance of Rotational Barriers: the Ionization of Meldrum's Acid. *J. Am. Chem. Soc.* **1987,** *109* (3), 809-812.

15. Kolthoff, I. M.; Chantooni, M. K., Substituent effects on dissociation of benzoic acids and heteroconjugation of benzoates with p-bromophenol in acetonitrile, N,N-dimethylformamide, and dimethyl sulfoxide. Intramolecular hydrogen bonding in o-hydroxybenzoic acids and their anions. *J. Amer. Chem. Soc.* **1971,** *93* (16), 3843-3849.
16. Nagai, H.; Kuwabara, K.; Carta, G., Temperature Dependence of the Dissociation Constants of Several Amino Acids. *J. Chem. Eng. Data* **2008,** *53* (3), 619-627.
17. Davies, C. W., 397. The extent of dissociation of salts in water. Part VIII. An equation for the mean ionic activity coefficient of an electrolyte in water, and a revision of the dissociation constants of some sulphates. *J. Chem. Soc.* **1938,** (0), 2093-2098.
18. Wallace, M.; Adams, D. J.; Iggo, J. A., Titrations without the Additions: The Efficient Determination of pKa Values Using NMR Imaging Techniques. *Anal. Chem.* **2018,** *90* (6), 4160-4166.
19. Liu, M.; Mao, X.-a.; Ye, C.; Huang, H.; Nicholson, J. K.; Lindon, J. C., Improved WATERGATE Pulse Sequences for Solvent Suppression in NMR Spectroscopy. *Journal of Magnetic Resonance* **1998,** *132* (1), 125-129.
20. Craven, M.; Yahya, R.; Kozhevnikova, E. F.; Robertson, C. M.; Steiner, A.; Kozhevnikov, I. V., Alkylaminophosphazenes as Efficient and Tuneable Phase-Transfer Agents for Polyoxometalate-Catalysed Biphasic Oxidation with Hydrogen Peroxide. *ChemCatChem* **2016,** *8* (1), 200-208.
21. Matthews, W. S.; Bares, J. E.; Bartmess, J. E.; Bordwell, F. G.; Cornforth, F. J.; Drucker, G. E.; Margolin, Z.; McCallum, R. J.; McCollum, G. J.; Vanier, N. R., Equilibrium acidities of carbon acids. VI. Establishment of an absolute scale of acidities in dimethyl sulfoxide solution. *Journal of the American Chemical Society* **1975,** *97* (24), 7006-7014.
22. Cox, B., Determination of Dissociation Constants. In *Acids and bases: solvent effects on acid-base strength*, Oxford University Press: Oxford, 2013.
23. Kütt, A.; Leito, I.; Kaljurand, I.; Sooväli, L.; Vlasov, V. M.; Yagupolskii, L. M.; Koppel, I. A., A Comprehensive Self-Consistent Spectrophotometric Acidity Scale of Neutral Brønsted Acids in Acetonitrile. *J. Org. Chem.* **2006,** *71* (7), 2829-2838.
24. Chalaris, M.; Marinakis, S.; Dellis, D., Temperature effects on the structure and dynamics of liquid dimethyl sulfoxide: A molecular dynamics study. *Fluid Phase Equilib.* **2008,** *267* (1), 47-60.
25. Valencia, D. P.; González, F. J., Estimation of diffusion coefficients by using a linear correlation between the diffusion coefficient and molecular weight. *J. Electroanal. Chem.* **2012,** *681*, 121-126.
